# Semi-mechanistic population pharmacokinetic/pharmacodynamic modeling of a *Plasmodium* elongation factor 2 inhibitor cabamiquine for prevention and cure of malaria

**DOI:** 10.1128/aac.00891-23

**Published:** 2023-11-15

**Authors:** Perrine Courlet, Justin J. Wilkins, Claude Oeuvray, Wei Gao, Akash Khandelwal

**Affiliations:** 1 Merck Institute for Pharmacometrics, Ares Trading S.A., (an affiliate of Merck KGaA, Darmstadt, Germany), Lausanne, Switzerland; 2 Occams Coöperatie UA, Amstelveen, the Netherlands; 3 The Global Health Institute of Merck (an affiliate of Merck KGaA, Darmstadt, Germany), Eysins, Switzerland; 4 EMD Serono Research and Development Institute, Inc., Billerica, Massachusetts, USA; 5 The healthcare business of Merck KGaA, Darmstadt, Germany; University Children's Hospital Münster, Münster, Germany

**Keywords:** malaria, dose–response, model-based drug development

## Abstract

Cabamiquine is a novel antimalarial agent that demonstrates the potential for chemoprevention and treatment of malaria. In this article, the dose–exposure–response relationship of cabamiquine was characterized using a population pharmacokinetic (PK)/pharmacodynamic (PD) model, incorporating the effects of cabamiquine on parasite dynamics at the liver and blood stages of malaria infection. Modeling was performed sequentially. First, a three-compartmental population PK model was developed, comprising linear elimination, a transit absorption model in combination with first-order absorption, and a recirculation model. Second, this model was expanded into a PK/PD model using parasitemia data from an induced blood stage malaria (IBSM) human challenge model. To describe the parasite growth and killing in the blood, a turnover model was used. Finally, the liver stage parasite dynamics were characterized using data from a sporozoite challenge model (SpzCh), and system parameters were fixed based on biological plausibility. Cabamiquine concentration in the central compartment was used to drive parasite killing at the blood and liver stages. Blood stage minimum inhibitory concentrations (MIC_b_) were estimated at 7.12 ng/mL [95% confidence interval (CI_95%_): 6.26–7.88 ng/mL] and 1.28 ng/mL (CI_95%_: 1.12–1.43 ng/mL) for IBSM and SpzCh populations, respectively, while liver stage MIC_l_ was lower (0.61 ng/mL; CI_95%_: 0.24–0.96 ng/mL). In conclusion, a population PK/PD model was developed by incorporating parasite dynamics and drug activity at the blood and liver stages based on clinical data and biological knowledge. This model can potentially facilitate antimalarial agent development by supporting the efficient selection of the optimal dosing regimen.

## INTRODUCTION

Parasite species of the genus *Plasmodium* are responsible for human malaria. Worldwide, this disease continues to cause an acute, debilitating illness associated with high mortality (1). *Plasmodium falciparum* is the most common and lethal malarial parasite, and if prompt and appropriate treatment is not received, it is associated with a high risk of complications and death (2, 3). Although several established treatments are available to treat *falciparum* malaria, the constantly evolving resistance to these treatments has resulted in a continuous need for the development of new antimalarial drugs (4, 5). Additionally, a cornerstone of malarial control is targeting the asymptotic liver stage infection through chemoprevention. However, available treatments present challenges (such as adverse effects and adherence to daily dosing regimens), again emphasizing the need for developing new drugs to prevent infection.

Cabamiquine (previously known as DDD107498 or M5717) is a new investigational compound that exhibits potent antimalarial activity against multiple life-cycle stages of the parasite. The molecular target of cabamiquine is the eukaryotic translation elongation factor 2 (eEF2), which is responsible for the guanosine triphosphate-dependent translocation of the ribosome along mRNA and is therefore essential for protein synthesis (6, 7). With a long half-life [146 h–193 h at doses ≥200 mg (8)] and activity against the liver stage (9), cabamiquine has the potential to address several clinical needs in malaria chemotherapy, including single-dose treatment and transmission blocking via inhibition of gametocyte formation, and chemoprevention. It has demonstrated good pharmacokinetic (PK) and pharmacodynamic (PD) properties and an acceptable safety profile in preclinical studies, animal models (6, 7), and in a recently completed combined phase Ia and Ib experimental infection study in healthy subjects (8). The development of cabamiquine in the chemoprotection indication requires a good understanding of the effect of the molecule against all stages of malaria infection. However, the lack of direct measurement of liver-stage parasites makes the evaluation of the level of chemoprotection challenging. In this context, model-informed approaches can help generate evidence on the unobserved parasite dynamics and drug effects at the liver stage by combining clinical PK and blood-stage parasite data, as well as biological knowledge on the parasite life-cycle.

In this study, we characterized the dose–exposure–parasite killing relationship for cabamiquine at blood and liver stages via semi-mechanistic population PK/PD modeling.

## MATERIALS AND METHODS

### Data set

**Fig 1:**
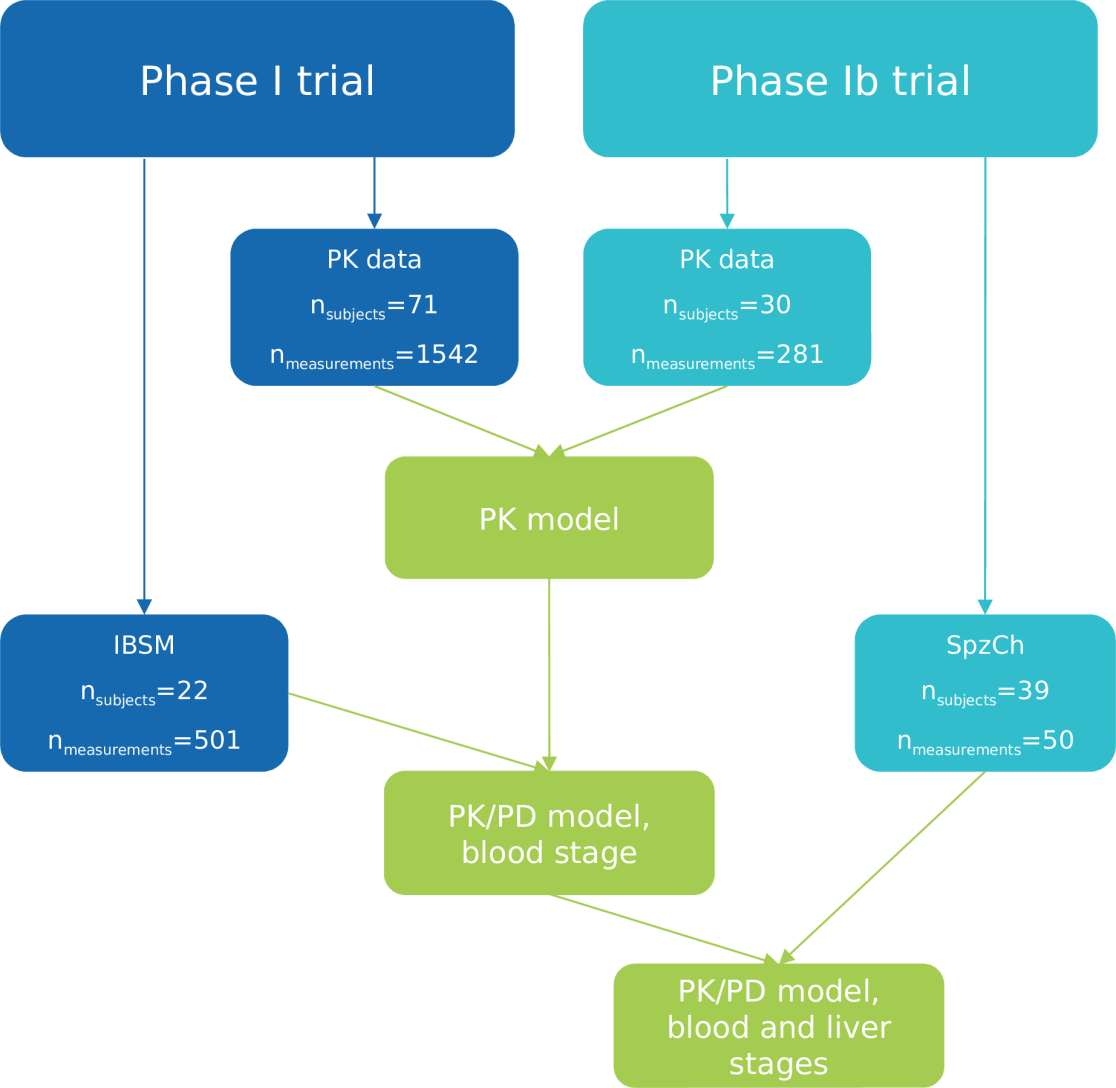
Flow chart summarizing the data available to inform each modeling step. IBSM: induced blood stage malaria, PD: pharmacodynamics, PK: pharmacokinetics, SpzCh: sporozoite challenge.

Data are summarized in Fig. 1. Cabamiquine blood concentrations and blood-stage parasitemia are pooled from two phase I trials.

The first study was a phase I, first-in-human trial, in which the safety, tolerability, PK profile of oral doses of cabamiquine, and its activity against *P. falciparum* were assessed in fasted healthy adult subjects (NCT03261401) (8). The trial was divided into two parts. Part 1 was a double-blind, randomized, placebo-controlled, single ascending dose study consisting of nine dose cohorts. Part 2 was a *P. falciparum*-induced blood stage malaria model (IBSM) where healthy volunteers were inoculated with infected erythrocytes (strain 3D7) 8 days before cabamiquine administration. Healthy adult men (aged 18–55 years) were included in the study. In this first study, cabamiquine was administered as succinate salt (1 mg of succinate salt is equivalent to 0.797 mg free base).

The second study was a phase Ib, randomized, double-blind, placebo-controlled, sequential study of single oral doses of cabamiquine conducted to explore its chemoprophylactic activity in a controlled sporozoite challenge model (SpzCh) in healthy participants (10). Participants received cabamiquine at different doses (30 mg, 60 mg, 80 mg, 100 mg, and 200 mg free base) or placebo in early and late liver stages, with the drug administered 2 h or 96 h after inoculation of approximately 3,200 sporozoites (strain NF54), respectively.

All participants provided written informed consent before the study trial procedures were initiated. Studies were approved by QIMR Berghofer Medical Research Institute Human Research Ethics Committee (8) and by the independent medical ethics committee Stichting Beoordeling Ethiek Biomedisch Onderzoek (Assen, The Netherlands) (10).

Parasitemia was measured using quantitative polymerase chain reaction (qPCR) with a lower limit of quantification (LLOQ) of 1 parasite/mL.

### Model development

#### Population PK modeling

A population PK model for cabamiquine was developed using data from the two phase I trials. The two- and three-compartment structures, various models for absorption and recirculation, different inter-individual variability (IIV) parameterizations, and potential sources of dose-nonproportionality were explored. The absorption models considered comprised of a first-order process, inclusion of a recirculation model to describe the secondary peak, and a flexible transit compartment model, to reflect the highly variable absorption observed during graphical analysis.

#### Population PK/PD modeling

##### Blood-stage modeling

**Fig 2:**
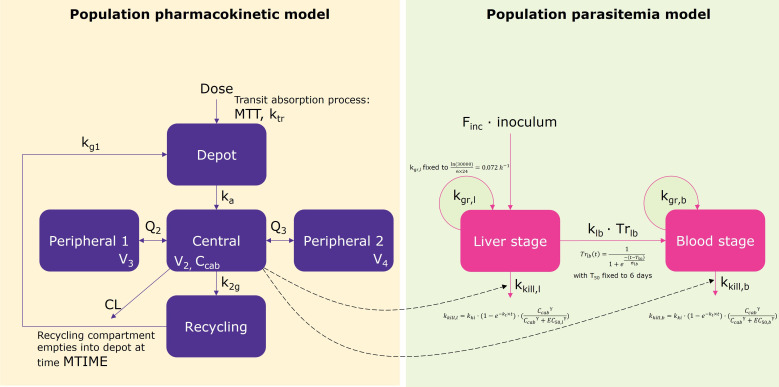
Schematic representation of the PK/PD model at blood and liver stages. CL, clearance; C_cab_, cabamiquine concentration in the central compartment; k_a_, absorption rate constant; k_g1_, depot to absorption transfer rate constant; k_2g_, central to depot transfer rate constant; k_tr_, transit rate between compartments; MTIME, depot emptying time; MTT, mean transit time; Q_2_, intercompartmental clearance 1; Q_3_, intercompartmental clearance 2; V_2_, central volume of distribution; V_3_, peripheral volume of distribution 1; V_4_, peripheral volume of distribution 2. EC_50,b_, cabamiquine concentration at which half of the maximum effect is attained at the blood stage; EC_50,l_, cabamiquine concentration at which half of the maximum effect is attained at the liver stage; F_inc_, fraction of infectious inoculated sporozoites that invade hepatocytes; Inoculum, number of injected sporozoites; k_gr,b_, parasite growth rate in blood; k_gr,l_, parasite growth rate in liver; k_ki_, parasite killing rate; k_lb_, rate of merozoites invading erythrocytes once they leave hepatocytes; k_t_, delay rate constant; Tr_lb_, cumulative fraction of hepatocytes that burst over time; T_50_, time by which half the hepatocytes burst after inoculation; γ, Hill coefficient; σ_lb_, the distribution of release around T_50_.

To incorporate the effect of cabamiquine on parasite killing, the population PK model was expanded using the parasitemia data from the IBSM study. Individual PK parameter estimates were carried forward into the PKPD model. Owing to a low number of subjects, the high variability in the data, and the need to support the straightforward derivation of efficacy parameters, a simple turnover model was used to describe the parasite dynamics at the blood stage, similar to the approach adopted by Krause et al. (11). The model was defined in terms of the parasite growth (k_growth_) and killing (k_ki_; Fig. 2) rate constants, with IIV being assessed on all structural parameters. An E_max_ model was used to characterize the drug-mediated killing effect of parasites. The Hill coefficient was fixed to a value obtained during previous analyses (12). To handle the delay in the onset of effect in the first 48 h as evidenced by visual exploration of parasitemia data, an exponential function was tested (Fig. 2), to allow killing rates to maximize over time.

##### Joint blood- and liver-stage modeling

The liver-stage compartment was added to the blood-stage model using data from SpzCh study and the following methodology. System parameters were fixed based on biological knowledge (13) (i.e., the transfer function from the liver to blood, parasite growth rate constant in liver k_gr,l_, and a fraction of inoculated sporozoites invading hepatocytes F_inc_). Assuming exponential growth and considering that 30,000 merozoites are released by each infected hepatocyte after 6 days, k_gr,l_ was calculated to be 0.072/h (Fig. 2). A previously reported transfer function (13) was used to characterize the release of merozoites from the liver to blood. Briefly, this function assumes a quick invasion of erythrocytes by merozoites (10 min) and a release of the major part of merozoites at day 6 ± 30 min (Fig. 2). Considering the limited placebo data available from the SpzCh study, F_inc_ was fixed to a previously reported value (13) corresponding to a typical value of four infected hepatocytes with IIV. The blood volume was assumed to be 5 L for all subjects (14). Additionally, some parameters estimated during blood-stage modeling were fixed (i.e.*,* baseline parasite count in the IBSM study P_0_ with IIV, parasite growth rate constant in blood k_gr,b_ with IIV, parasite killing rate constant k_ki_ with IIV, delay rate constant k_t_, and Hill coefficient γ) to allow precise estimation of the drug effect parameter on the liver. Several parametrizations were compared in terms of shared parameters between blood- and liver-stage models, and IIV.

##### Derivation of key efficacy parameters

From the final PD models, the minimum inhibitory concentration (MIC) and the concentration at which the parasite killing effect is at 90% of the maximum (minimum parasiticidal concentration, MPC_90_) at both blood and liver stages were derived as follows (15):


$$MIC=EC_{50}\cdot {\left(\frac{k_{gr}}{k_{ki}-k_{gr}}\right)}^{\frac{1}{\gamma }}$$

$$MPC_{90}=9^{\frac{1}{\gamma }}\cdot EC_{50}$$

where k_gr_ denotes the parasite growth rate in blood or liver, EC_50_ is the drug concentration required to produce 50% of the maximal killing rate in blood or liver, k_ki_ denotes the parasite killing rate, and γ denotes a shape parameter describing the shape of the relationship.

### Software

R (version 4.1.1) was used for exploratory analyses, data management, and post-processing steps. Population PK and PK/PD modeling were performed using the nonlinear mixed-effect modeling software Monolix (version 2021R2) with the stochastic approximation expectation–maximization (SAEM) estimation algorithm. Data below the lower limit of quantification (BLOQ) were handled using the M3 method (16). Model-based simulations were conducted using Simulx (version 2021R2). All computational software was installed in a validated GxP environment.

### Model qualification

Models were evaluated using standard criteria, including objective function value (OFV), goodness-of-fit plots, and the uncertainty and plausibility of parameter estimates. Given the low number of individuals and the relative complexity of the models, their robustness and their sensitivity to initial conditions were evaluated using the convergence assessment tool in Monolix. Additionally, visual predictive checks (VPCs) were used to evaluate the predictive performances of the models by comparing observations with 90% prediction intervals derived from 500 simulations (17).

### Model evaluation

The model was evaluated by comparing the observed and predicted success rates across dose groups, based on the conditions of the trials (i.e., design and sample size). The Clopper–Pearson interval was used to compute the CI_95%_ of the observed success rates. To estimate the predicted success rate, simulated participants with parasitemia below 1 parasite in the body (i.e.*,* 1 p/5,000 mL) were not allowed to recrudesce and were considered cured for the remaining simulation time (18). This was used as a pragmatic approach to avoid implausible recrudescence simply attributed to the exponential nature of the growth phase since such a low parasitemia threshold would certainly not lead to recrudescence in clinical practice owing to the response of the immune system. Success rates were then defined as follows:

1. IBSM study: percentage of subjects without recrudescence (defined as >5,000 parasites/mL and ≥two-fold increase within 48 h)
2. SpzCh study: percentage of participants without positive parasitemia (defined as first positive qPCR outcome of ≥100 parasites/mL of blood within 28 days of SpzCh)

Virtual trials were replicated 1,000 times, and medians and CI_95%_ values of predicted success rates were computed for each treatment group.

## RESULTS

### PK/PD data

**Fig 3:**
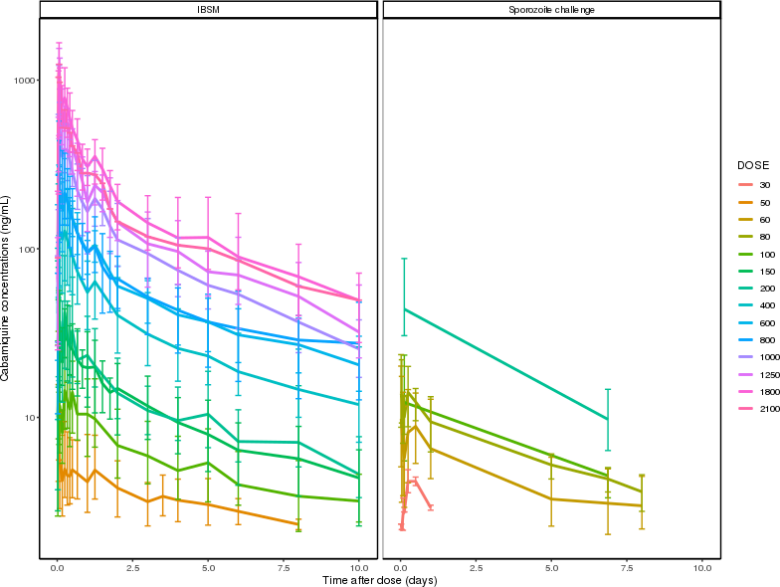
Geometric mean concentration–time profiles of cabamiquine by study and dose group until day 10. Error bars show 95% confidence intervals (assessed as the 2.5th and 97.5th quantiles by nominal time point). IBSM: induced blood stage malaria.

The PK data set comprised 1,823 evaluable cabamiquine concentrations from 101 healthy subjects across 14 doses (Fig. 1). The PK profile of cabamiquine was complex, showing greater than dose proportionality, highly variable absorption, and secondary peaks (Fig. 3).

**Fig 4:**
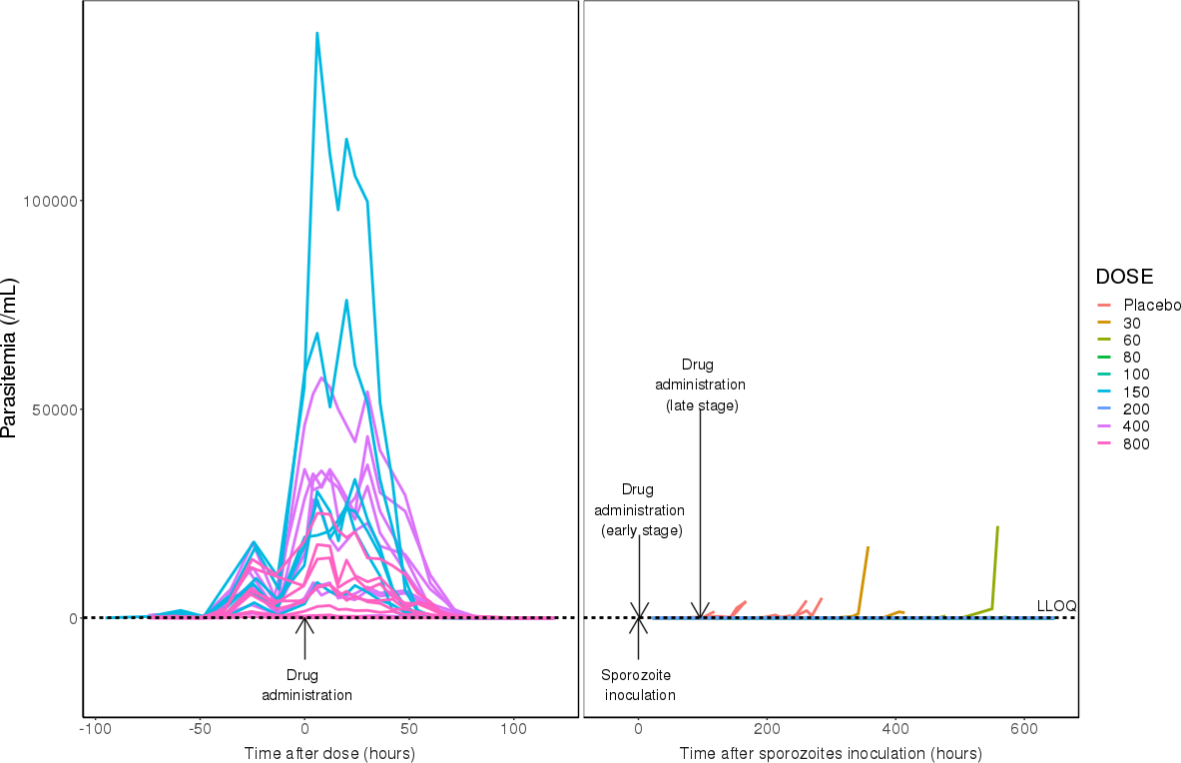
Individual parasitemia profiles over time in the IBSM subjects between 96 h pre-dose and 120 h post-dose (left panel) and in the SpzCh subjects (right panel). LLOQ: lower limit of quantification.

For blood-stage modeling, 501 parasitemia measures across three dose levels (150 mg, 400 mg, and 800 mg, succinate salt) were evaluated from 22 participants in the IBSM challenge model (Fig. 1). Parasitemia time profiles from IBSM subjects are shown in Fig. 4 (left panel). This plot revealed several noteworthy features in parasitemia data. As previously reported (8), the parasite life cycle was highly synchronous in all subjects, with a regular dip in parasite level coinciding with sequestration of parasites during the latter half of the life cycle. Close examination of the pre-dose parasite growth revealed that although the growth rate was consistent across all subjects, there were profound differences in the extent of parasitemia at the time of dosing. Additionally, as previously reported (8), the decline of parasitemia after dose administration exhibited a biphasic pattern, with the first phase occurring from 6 h to 48 h after cabamiquine dosing, followed by a second phase (the main killing phase). Killing of parasites was observed in all subjects following cabamiquine treatment. Recrudescent parasitemia occurred in three of the six subjects in the 150 mg group (50%) and two of the eight subjects in the 400 mg group (25%), but no recrudescence was observed in the 800 mg group.

The liver-stage modeling was developed based on 50 evaluable parasitemia data from 39 participants receiving placebo (*n* = 9) or cabamiquine (*n* = 30) across eight dose levels (30 mg, 60 mg, 80 mg, 100 mg, 150 mg, 200 mg, 400 mg, and 800 mg; free base) (Fig. 1). Parasitemia time profiles from SpzCh subjects are shown in Fig. 4 (right panel). This plot highlights the delay in parasitemia appearance in a dose-dependent manner and complete protection with 100 mg and 200 mg cabamiquine.

Overall, 60% of parasitemia measures were below the LLOQ, most of them (72%) being collected in the SpzCh study.

### Population PK modeling

The final parsimonious population PK model had three compartments, with linear elimination, a transit absorption model in combination with first-order absorption, and a recirculation model to account for a secondary peak between 24 h and 30 h. In the recirculation model, the drug was allowed to collect in a depot compartment before being released as a bolus back into the absorption compartment, with a strong inverse relationship between the central volume of distribution and dose (with a power coefficient of −0.530; Supplementary Material 1). Several attempts were made to account for the observed lack of dose-proportionality in the PK data, and it became clear that the issue lay with distribution rather than clearance. Various mechanistic attempts to explain the observations in terms of (more) complex absorption and tissue binding failed, and it was ultimately necessary to include an empirical covariate effect of dose on V_2_/F in the model. The rate of transfer of cabamiquine into the depot recirculation compartment (k_g1_) was fixed to improve the model’s stability and allow reasonable uncertainty estimates for the model parameters. IIV was estimated on apparent clearance (CL/F), apparent central volume of distribution (V_2_/F), bioavailability (F) and absorption rate (k_a_), transit rate between compartments (k_tr_), mean transit time (MTT), and central to depot transfer rate constant (k_2g_). Allometric scaling was applied to clearance and volume parameters (19). Standard diagnostic goodness-of-fit plots revealed no unacceptable trends, and VPCs evidenced the good predictive performances of the model (Supplementary Material 2). Therefore, the population PK model was judged to be acceptable for use in further PK/PD model development.

### Population PK/PD modeling

#### Blood-stage modeling

High IIV was estimated on the baseline parasitemia parameter [coefficient of variation (CV%) of IIV of 197], consistent with graphical explorations. However, and as previously reported (8), the parasite growth rate was consistent across all subjects, with a lower IIV estimated on this parameter CV% 12). IIV was also estimated on the blood-stage EC_50_ (EC_50,b,IBSM_) and the growth and killing rate constants (k_gr,b_ and k_ki_, respectively). The biphasic pattern of parasitemia decline was described through an inverse-exponential model with a typical half-life of 23.1 h before maximal killing.

In the final blood-stage model, parameters were precisely estimated [relative standard error (RSE) ≤42%; Supplementary Material 3], and VPCs showed close alignment between observations and predictions (Supplementary Material 4). Individual plots showed that the model could describe and predict recrudescence adequately (data not shown). Shrinkage was also high across all IIV parameters for the parasitemia turnover model, likely a reflection of the relatively small number of subjects (data not shown).

#### Joint blood- and liver-stage modeling

**Fig 5:**
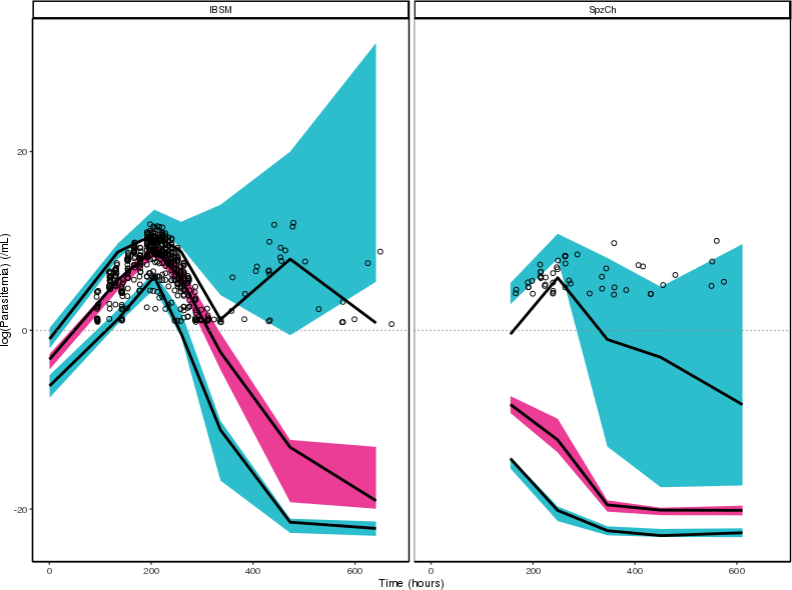
Visual predictive check for the joint blood- and liver-stage model. Points are observations. Solid lines are median observations and 90% intervals for observations. Pink- and blue-shaded areas are 90% intervals for the predicted median and 90% intervals for 90% ranges of observations, respectively. The gray dotted line shows the LLOQ. IBSM: induced blood stage malaria, SpzCh: sporozoite challenge.

The same structural model was used to describe parasite life cycle and drug activity at blood and liver stages. Killing and delay rate constants (k_ki_ and k_t_, respectively) were assumed to be similar for blood and liver to allow for the estimation of the liver stage EC_50_ (EC_50,l_) with a robust convergence. A 100% correlation was assumed between the growth rate constants in blood and liver (k_gr,b_ and k_gr,l_, respectively). The limited data from SpzCh study prevented the estimation of a dedicated k_gr,b_ for this population to account for potential differences between the two parasite strains. Nevertheless, our value was in fair concordance with the one reported in the literature (13). In the first attempts, we estimated a shared EC_50,b_ parameter for the IBSM and SpzCh populations. However, this resulted in a very low estimation of EC_50,b_, not comparable with that obtained in the blood-stage model. Graphical explorations of these results revealed that this low parameter estimate was mainly driven by individuals from the SpzCh study, with higher values estimated for patients from IBSM study. Therefore, we estimated separate blood-stage EC_50_ for the IBSM and SpzCh studies (i.e., EC_50,b,IBSM_ and EC_50,b,SpzCh_, respectively), resulting in lower OFV and improved VPCs. The estimation of IIV on EC_50,l_ was not supported by the data, which likely reflects the relatively low number of subjects and the large proportion of BLOQ data in the SpzCh population. Table 1 shows the parameter estimates of the final joint blood and liver stages model. The VPCs shown in Fig. 5 illustrate that the joint PK/PD model adequately regenerated the parasitemia data in both study populations, although we observed a trend for overprediction in the initial phase of SpzCh parasitemia profile. Further exploration revealed that this was mainly attributed to the placebo group, although the low number of subjects in each dose group prevented the correct interpretation of VPCs stratified by dose. Individual plots for all subjects showed reasonable agreement between the observed and predicted parasitemia profiles.

**TABLE 1:** Parameter estimates for the joint blood- and liver-stage model

Parameter	Estimate	Relative standard error (%)
Baseline parasitemia (P_0_, parasites/mL)^*a*^	0.03	
Parasite killing rate (k_ki_ /h)^*a*^	0.21	
EC_50,b,IBSM_ (ng/mL)	7.60	10.2
EC_50,b,SpzCh_ (ng/mL)^*c*^	1.29	
EC_50,l_ (ng/mL)	0.66	19.9
Parasite growth rate (k_gr,b_ /h)^*a*^	0.064	
Hill coefficient (γ)^*a*^	19	
Delay rate constant (k_t_ /h)^*a*^	0.030	
Rate of merozoites invading erythrocytes (k_lb_ /h)^*b*^	6	
Time by which half the hepatocytes burst after infection (T_50_, h)^*b*^	144	
Distribution of release around time (σ_lb_)^*b*^	0.1	
Parasite growth rate in liver (k_gr,l_)^*b*^	0.072	
Fraction of infectious inoculated sporozoites invading hepatocytes (F_inc_)^*b*^	0.0012	
IIV on P_0_^*a*^	197	
IIV on k_ki_^*a*^	19	
IIV on EC_50,b,IBSM_	45	16.8
IIV on k_gr,b_^*a*^	12	
IIV on k_gr,l_^*a*^	11	
IIV on F_inc_^*b*^	14	
Correlation between k_gr,b_ and k_gr,l_	1	Fixed
Residual error [parasitemia, log(/mL)]	1.68	3.30

^*a*^ Parameters fixed to the values estimated during blood-stage modeling.

^*b*^ Parameters fixed based on literature data (13)

^*c*^ Equation of the effect of SpzCh study based on EC_50,b,IBSM_: *EC*_50,*b*,*SpzCh*_ = *EC*_50,*b*,*IBSM*_ *×* (1−*θ × SpzCh*) with SpzCh being equal to 0 or 1 for IBSM and SpzCh populations, respectively. The effect parameter (i.e., θ) was estimated to be 0.83 with RSE 0.4%.

#### Derivation of key efficacy parameters

Table 2 summarizes MIC and MPC_90_ values derived from PK/PD models and shows that EC_50,b_ values for IBSM were similar across models. Overall, MIC and MPC_90_ were lower for the SpzCh population than for the IBSM population, and the parameters were lower in the liver than in the blood.

**TABLE 2:** Comparison of key efficacy parameters derived from different models^*a*^

Parameter	Blood-stage model	Joint blood- and liver-stage model
MIC_b,IBSM_	7.51 (6.72–8.25)	7.12 (6.26–7.88)
MIC_b,SpzCh_		1.28 (1.12–1.43)
MIC_l_		0.61 [0.24–0.96]
MPC_90,b,IBSM_	8.84 (8.52–9.15)	8.35 (8.12–8.56)
MPC_90,b,SPzCh_		1.50 (1.44–1.57)
MPC_90,l_		0.70 (0.28–1.11)

^*a*^ Results are presented as median (CI_95%_), computed using variance–covariance matrix to reflect the uncertainty of parameter estimates.

### Model evaluation

**Fig 6:**
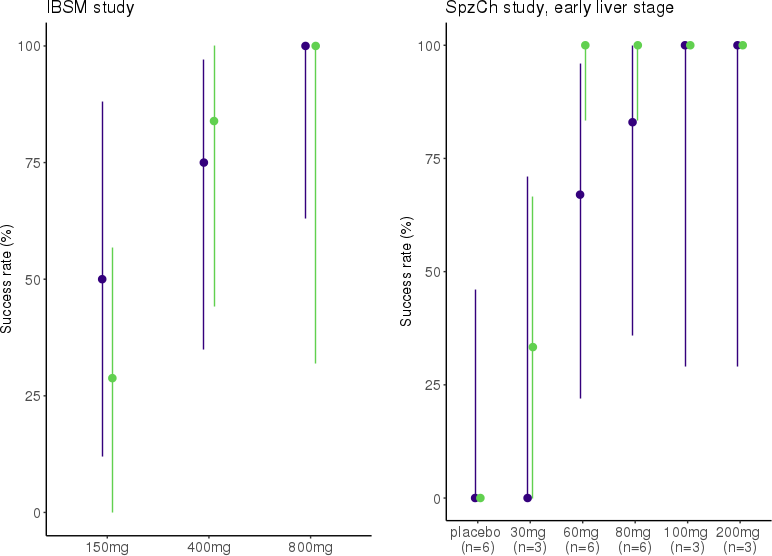
Observed (purple) and predicted (green) success rates for IBSM and SpzCh populations. Medians are represented with points and 95% confidence intervals with bars. IBSM: induced blood stage malaria, SpzCh: sporozoite challenge.

Despite a large variability likely due to the small number of participants, model-based simulations highlighted the adequate predictive performances of the model. Predicted CI_95%_ of success rates largely overlapped the observed ones, as evidenced in Fig. 6. Results should be interpreted with caution because success rates are impacted by the low sample size in each dose group.

## DISCUSSION

In this study, we developed a semi-mechanistic population PK/PD model to characterize cabamiquine’s killing effect on parasites at the blood and liver stages.

The final PK model designed for this investigation reflected the complex and nonlinear distribution processes of cabamiquine. The presence of secondary peaks in the PK profile of cabamiquine, notably at 6–8 h and 24–30 h post-dosing, which loosely correspond to mealtimes, suggest enterohepatic recycling, a process wherein xenobiotics entering the alimentary tract are absorbed and then re-excreted into the bile to be re-deposited in the intestinal lumen for repeat absorption (20). As observed for cabamiquine, drugs subject to enterohepatic recycling are often characterized by a long half-life and multiple concentration peaks. A recycling model was useful for characterizing the secondary peaks at 24–30 h post-dose. The PK model was as semi-mechanistic as could be supported by the data; however, absorption, distribution, and recycling are the result of related processes, and once these processes are better understood and more data are available, it may be possible to develop a more physiologically plausible model. Despite these limitations, this PK model was deemed to be suitable and was successfully used in combination with *in vitro* data to predict the starting dose to be used in the SpzCh study through model-based simulations (9).

The PK model provided the starting point for a joint PK/PD model that describes the effect of drug concentration on parasitemia profiles at blood and liver stages of malaria infection. Although the final model simplified the complex underlying process related to the PK/PD of cabamiquine in humans, it enabled an investigation of the dose–exposure–parasite killing relationship via modeling. Considering the limited number of subjects and the underlying complexity of the system, an empirical approach was used to describe the concentration-effect relationship of cabamiquine. This model was considered to be appropriate and improved our understanding of the concentration–effect relationship of cabamiquine. Owing to the lack of direct measurement of parasites in the liver, the structural model of the blood stage had to be borrowed and completed based on biological knowledge about parasite life cycle to infer parasites’ dynamics in the liver. The challenge of characterizing drug activity at the liver stage is common to all antimalaria clinical programs. Nevertheless, by leveraging semi-mechanistic modeling to integrate quantitative data and qualitative knowledge, such population PK/PD analyses can potentially support clinical drug development.

In the final model, MIC_b_ was estimated to be lower in the SpzCh population than in the IBSM population (EC_50,b_ values of 1.28 ng/mL and 7.12 ng/mL, respectively). This difference may arise from the effect of cabamiquine at the liver stage, not only reducing the density of parasites reaching blood but also affecting their viability (21). We hypothesized that cabamiquine killed and damaged parasites in the liver, thus reducing the survival of the merozoites released thereafter into the bloodstream. Thus, lower drug concentrations might be required to kill such drug-damaged parasites. Further studies are needed to confirm this hypothesis. MIC_l_ was estimated at a lower value of 0.61 ng/mL, reflecting the superior efficacy of cabamiquine in the liver than in the blood. This is consistent with previously published results wherein the pre-erythrocytic activity of cabamiquine was demonstrated *in vitro* (6)*,* and with the observed clinical data in healthy volunteers requiring lower doses for chemoprevention than cure.

Overall, we confirmed that it is possible to infer information about drug effects at the liver stage by combining prior knowledge about parasite life cycle and clinical data in a mathematical model. The integration of data from two studies with different designs allowed the assessment of drug effects at the blood and liver stages. Such a methodology can potentially support the development of antimalarial compounds by providing a better understanding of drug effects at different stages of the parasite life cycle. Future extensions of this study involve model-based simulations to predict efficacy in the cure and chemoprophylactic settings. By comparing several scenarios in terms of timing and levels of infections, such simulations have value in enabling a more efficient selection of appropriate dosing regimens, thereby increasing the probability of success in later development phases in this critical therapeutic area. The model can also be improved to include re-infection scenarios with multiple mosquito bites, better reflecting a real-world setting. Finally, to evaluate the impact of administering cabamiquine in malaria-endemic regions, PK and PD parameters can be embedded within malaria transmission models, taking into consideration different transmission patterns, treatment access, and health system costs (22).

This study has some limitations. Parasitemia data were derived from a relatively small and homogenous population of 61 healthy, malaria-naïve adult Caucasian men. Therefore, the results reported herein should be viewed in this context (23), and the model may require to be updated when data from a patient population are available to better understand the similarity or otherwise of PK/PD parameters between healthy malaria-naïve subjects and patients with variable immunity. Furthermore, drug effects were evaluated when administered in monotherapy. However, combinations of antimalarial drugs with different targets or resistance mechanisms should be administered to delay the development of *de novo* resistance in a real-world setting, in order to prolong their effective therapeutic life, as recommended by the World Health Organization (24). Although the combination should retain high antimalarial efficacy, the components of the combination should also provide mutual protection against selection for resistance to the individual components (25). In this context, a pre-clinical study was conducted to evaluate the parasitological behavior and PK parameters of cabamiquine and pyronaridine when used in combination (4). The results demonstrated the absence of PK interaction and an additive parasitological effect of the two compounds. Further *in vitro* and *in vivo* studies have been conducted to validate pyronaridine as a suitable partner drug for cabamiquine as a chemoprevention treatment, which supports the clinical evaluation of this novel combination therapy (26).

### Conclusions

A population PK/PD model was successfully developed to characterize cabamiquine activity against the blood and liver stages of malaria infection. The model suggested a higher potency of cabamiquine at the liver stage compared to the blood stage, which is consistent with the observed clinical data. This framework provides a quantitative understanding of the antimalarial activity of cabamiquine and can be used to support dose selections and study designs for future cure and chemoprevention trials.

## Data Availability

Any requests for data by qualified scientific and medical researchers for legitimate research purposes will be subject to the Data Sharing Policy of the healthcare business of Merck KGaA, Darmstadt, Germany. All requests should be submitted in writing to the data sharing portal for the healthcare business of Merck KGaA, Darmstadt, Germany https://www.emdgroup.com/en/research/our-approach-to-research-and-development/healthcare/clinical-trials/commitment-responsible-data-sharing.html. When the healthcare business of Merck KGaA has a co-research, co-development, or co-marketing or co-promotion agreement, or when the product has been out-licensed, the responsibility for disclosure might be dependent on the agreement between parties. Under these circumstances, the healthcare business of Merck KGaA will endeavor to gain agreement to share data in response to requests.

## SUPPLEMENTAL MATERIAL

Supplemental figures and tables aac.00891-23-s0001.docx Supplemental material 1–4.

ASM does not own the copyrights to Supplemental Material that may be linked to, or accessed through, an article. The authors have granted ASM a non-exclusive, world-wide license to publish the Supplemental Material files. Please contact the corresponding author directly for reuse.

## References

[1] World Health Organization. 2022. World malaria report 2022. doi:10.1177/13505084221090632

[2] Centres for Disease Control. 2021 About malaria. Available from: https://www.cdc.gov/malaria/about/faqs.html

[3] Trampuz A, Jereb M, Muzlovic I, Prabhu RM. 2003. Clinical review: severe malaria. Crit Care 7:315–323. doi:10.1186/cc2183 12930555 PMC270697

[4] Rottmann M, Jonat B, Gumpp C, Dhingra SK, Giddins MJ, Yin X, Badolo L, Greco B, Fidock DA, Oeuvray C, Spangenberg T. 2020. Preclinical antimalarial combination study of M5717, a Plasmodium falciparum elongation factor 2 inhibitor, and pyronaridine, a hemozoin formation inhibitor. Antimicrob Agents Chemother 64:e02181-19. doi:10.1128/AAC.02181-19 32041711 PMC7179297

[5] White NJ. 2004. Antimalarial drug resistance. J Clin Invest 113:1084–1092. doi:10.1172/JCI21682 15085184 PMC385418

[6] Baragaña B, Hallyburton I, Lee MCS, Norcross NR, Grimaldi R, Otto TD, Proto WR, Blagborough AM, Meister S, Wirjanata G, et al.. 2015. A novel multiple-stage antimalarial agent that inhibits protein synthesis. Nature 522:315–320. doi:10.1038/nature14451 26085270 PMC4700930

[7] Baragaña B, Norcross NR, Wilson C, Porzelle A, Hallyburton I, Grimaldi R, Osuna-Cabello M, Norval S, Riley J, Stojanovski L, et al.. 2016. Discovery of a quinoline-4-carboxamide derivative with a novel mechanism of action, multistage antimalarial activity, and potent in vivo efficacy. J Med Chem 59:9672–9685. doi:10.1021/acs.jmedchem.6b00723 27631715 PMC5108032

[8] McCarthy JS, Yalkinoglu Ö, Odedra A, Webster R, Oeuvray C, Tappert A, Bezuidenhout D, Giddins MJ, Dhingra SK, Fidock DA, Marquart L, Webb L, Yin X, Khandelwal A, Bagchus WM. 2021. Safety, pharmacokinetics, and antimalarial activity of the novel Plasmodium eukaryotic translation elongation factor 2 inhibitor M5717: a first-in-human, randomised, placebo-controlled, double-blind, single ascending dose study and volunteer infection study. Lancet Infect Dis 21:1713–1724. doi:10.1016/S1473-3099(21)00252-8 34715032 PMC8612936

[9] Khandelwal A, Arez F, Alves PM, Badolo L, Brito C, Fischli C, Fontinha D, Oeuvray C, Prudêncio M, Rottmann M, Wilkins J, Yalkinoglu Ö, Bagchus WM, Spangenberg T. 2022. Translation of liver stage activity of M5717, a Plasmodium elongation factor 2 inhibitor: from bench to bedside. Malar J 21:151. doi:10.1186/s12936-022-04171-0 35570264 PMC9107587

[10] van der Plas JL, Kuiper VP, Bagchus WM, Bödding M, Yalkinoglu Ö, Tappert A, Seitzinger A, Spangenberg T, Bezuidenhout D, Wilkins J, Oeuvray C, Dhingra SK, Thathy V, Fidock DA, Smidt LCA, Roozen GVT, Koopman JPR, Lamers OAC, Sijtsma J, Schuijlenburg R van, Wessels E, Meij P, Kamerling IMC, Roestenberg M, Khandelwal A. 2022 A randomised, double-blind, placebo-controlled study evaluating the chemoprophylactic activity of M5717 against Plasmodium falciparum in a controlled human malaria infection. SSRN J. doi:10.2139/ssrn.4279600 37414066

[11] Krause A, Dingemanse J, Mathis A, Marquart L, Möhrle JJ, McCarthy JS. 2016. Pharmacokinetic/pharmacodynamic modelling of the antimalarial effect of actelion-451840 in an induced blood stage malaria study in healthy subjects. Br J Clin Pharmacol 82:412–421. doi:10.1111/bcp.12962 27062080 PMC4972157

[12] Wilkins J, Bagchus W, Oeuvray C, Khandelwal A. 2021. PK/PD modelling of M5717 in malaria. PAGE meeting.

[13] Cherkaoui-Rbati MH, Andenmatten N, Burgert L, Egbelowo OF, Fendel R, Fornari C, Gabel M, Ward J, Möhrle JJ, Gobeau N. 2023. A pharmacokinetic–pharmacodynamic model for chemoprotective agents against malaria. CPT Pharmacometrics Syst Pharmacol 12:50–61. doi:10.1002/psp4.12875 36412499 PMC9835136

[14] Sharma R, Sharma S. 2023. Physiology, blood volume. In StatPearls. StatPearls Publishing, Treasure Island (FL). https://www.ncbi.nlm.nih.gov/books/NBK526077/. 30252333

[15] McCarthy JS, Baker M, O’Rourke P, Marquart L, Griffin P, Hooft van Huijsduijnen R, Möhrle JJ. 2016. Efficacy of OZ439 (artefenomel) against early Plasmodium falciparum blood-stage malaria infection in healthy volunteers. J Antimicrob Chemother 71:2620–2627. doi:10.1093/jac/dkw174 27272721 PMC4992851

[16] Bergstrand M, Karlsson MO. 2009. Handling data below the limit of quantification in mixed effect models. AAPS J 11:371–380. doi:10.1208/s12248-009-9112-5 19452283 PMC2691472

[17] Bergstrand M, Hooker AC, Wallin JE, Karlsson MO. 2011. Prediction-corrected visual predictive checks for diagnosing nonlinear mixed-effects models. AAPS J 13:143–151. doi:10.1208/s12248-011-9255-z 21302010 PMC3085712

[18] Wicha SG, Walz A, Cherkaoui-Rbati MH, Bundgaard N, Kuritz K, Gumpp C, Gobeau N, Möhrle J, Rottmann M, Demarta-Gatsi C. 2022. New in vitro interaction-parasite reduction ratio assay for early derisk in clinical development of antimalarial combinations. Antimicrob Agents Chemother 66:e0055622. doi:10.1128/aac.00556-22 36197116 PMC9664866

[19] Anderson BJ, Holford NHG. 2008. Mechanism-based concepts of size and maturity in pharmacokinetics. Annu Rev Pharmacol Toxicol 48:303–332. doi:10.1146/annurev.pharmtox.48.113006.094708 17914927

[20] Roberts MS, Magnusson BM, Burczynski FJ, Weiss M. 2002. Enterohepatic circulation: physiological, pharmacokinetic and clinical implications. Clin Pharmacokinet 41:751–790. doi:10.2165/00003088-200241100-00005 12162761

[21] White NJ. 2017. Malaria parasite clearance. Malar J 16:88. doi:10.1186/s12936-017-1731-1 28231817 PMC5324257

[22] Okell LC, Cairns M, Griffin JT, Ferguson NM, Tarning J, Jagoe G, Hugo P, Baker M, D’Alessandro U, Bousema T, Ubben D, Ghani AC. 2014. Contrasting benefits of different artemisinin combination therapies as first-line malaria treatments using model-based cost-effectiveness analysis. Nat Commun 5:5606. doi:10.1038/ncomms6606 25425081 PMC4263185

[23] Bei AK, Ahouidi AD, Dvorin JD, Miura K, Diouf A, Ndiaye D, Premji Z, Diakite M, Mboup S, Long CA, Duraisingh MT. 2017. Functional analysis reveals geographical variation in inhibitory immune responses against a polymorphic malaria antigen. J Infect Dis 216:267–275. doi:10.1093/infdis/jix280 28605544 PMC5853457

[24] World Health Organization. n.d. Guidelines for the treatment of malaria. Available from: https://www.who.int/publications/i/item/guidelines-for-malaria

[25] Krishna S. 2019. Triple artemisinin-containing combination anti-malarial treatments should be implemented now to delay the emergence of resistance: the case against. Malar J 18:339. doi:10.1186/s12936-019-2976-7 31581951 PMC6777023

[26] Fontinha D, Arez F, Gal IR, Nogueira G, Moita D, Baeurle THH, Brito C, Spangenberg T, Alves PM, Prudêncio M. 2022. Pre-erythrocytic activity of M5717 in monotherapy and combination in preclinical Plasmodium infection models. ACS Infect Dis 8:721–727. doi:10.1021/acsinfecdis.1c00640 35312290 PMC9003234

