# Supplementary material for: Semi-mechanistic population pharmacokinetic/pharmacodynamic modeling of a Plasmodium elongation factor 2 inhibitor cabamiquine for prevention and cure of malaria
Source: Antimicrob Agents Chemother. 2023 Nov 15;67(12):e00891-23. doi: 10.1128/aac.00891-23 (PMC10720512; doi:10.1128/aac.00891-23)

# Supplementary Material

Supplementary Material 1: PK model parameter estimates

| Parameter | Estimate | Relative standard error (%) |
| --- | --- | --- |
| Clearance (CL/F, L/h) | 17.8 | 5.64 |
| Central volume of distribution (V_2_/F, L) | 2363 | 3.23 |
| Peripheral volume of distribution 1 (V_3_/F, L) | 2051 | 7.76 |
| Intercompartmental clearance 1 (Q_2_/F, L/h) | 6.37 | 0.0589 |
| Peripheral volume of distribution 2 (V_4_/F, L) | 2548 | 3.23 |
| Intercompartmental clearance 2 (Q_3_/F, L/h) | 58.5 | 3.08 |
| Absorption rate constant (k_a_ /h) | 8.27 | 21.7 |
| Central to depot transfer rate constant (k_2g_ /h) | 0.0039 | Fixed |
| Depot to absorption transfer rate constant (k_g1_ /h) | 15 | Fixed* |
| Transit rate between compartments (k_tr_ /h) | 13.7 | 5.66 |
| Mean transit time (MTT, h) | 0.21 | 11.6 |
| Depot emptying time (h) | 25.05 | 13.0 |
| Weight on CL/F | 0.75 | Fixed |
| Weight on V_2_/F | 1 | Fixed |
| Weight on V_3_/F | 1 | Fixed |
| Weight on Q_2_/F | 0.75 | Fixed |
| Weight on V_4_/F | 1 | Fixed |
| Weight on Q_3_/F | 0.75 | Fixed |
| Dose on V_2_/F | -0.50 | 5.18 |
| IIV on CL/F | 27 | 25.7 |
| IIV on V_2_/F | 25 | 10.2 |
| IIV on k_a_ | 340 | 10.5 |
| IIV on k_2g_ | 82 | 14.0 |
| IIV on k_tr_ | 25 | 18.1 |
| IIV on MTT | 110 | 9.78 |
| IIV on F | 23 | 9.28 |
| Residual error (%) | 17 | 1.88 |

IIV: inter-individual variability, reported as coefficient of variation (%)

*Fixed to a high number; transfer is near-instant.

Supplementary Material 2: Visual predictive check for the parsimonious PK model

Points are observations. Solid lines are median observations and 90% intervals for observations. Pink- and blue-shaded areas are 90% intervals for the predicted median and 90% intervals for 90% ranges of observations, respectively.


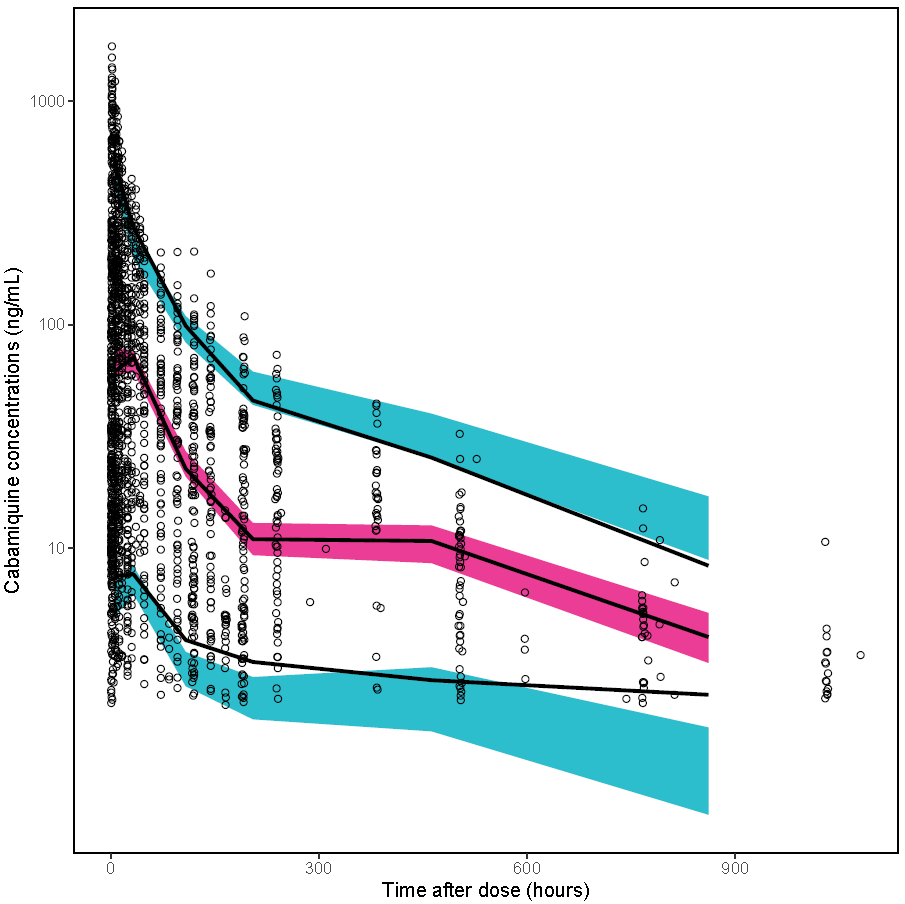


Supplementary Material 3: Blood-stage final model parameter estimates

| Parameter | Estimate | Relative standard error (%) |
| --- | --- | --- |
| Baseline parasitemia (P_0_, parasites/mL) | 0.03 | 41.3 |
| Parasite killing rate (k_ki_ /h) | 0.21 | 4.59 |
| EC_50,b,IBSM_ (ng/mL) | 8.35 | 14.4 |
| Parasite growth rate (k_gr,b_ /h) | 0.064 | 3.72 |
| Hill coefficient (γ) | 19.0 | Fixed |
| Delay rate constant (k_t_ /h) | 0.03 | 0.89 |
| IIV on P_0_ | 197 | 28.5 |
| IIV on k_ki_ | 19 | 16.7 |
| IIV on EC_50,b,IBSM_ | 51 | 20.3 |
| IIV on k_gr_ | 12 | 23.2 |
| Residual error (parasitemia, log(/mL)) | 1.46 | 3.44 |

IBSM: induced blood stage malaria, IIV: inter-individual variability, reported as coefficient of variation (%)

Supplementary Material 4: Visual predictive check for the blood-stage model.

Points are observations. Solid lines are median observations and 90% intervals for observations. Pink and blue shaded areas are 90% intervals for the predicted median and 90% intervals for 90% ranges of observations, respectively.


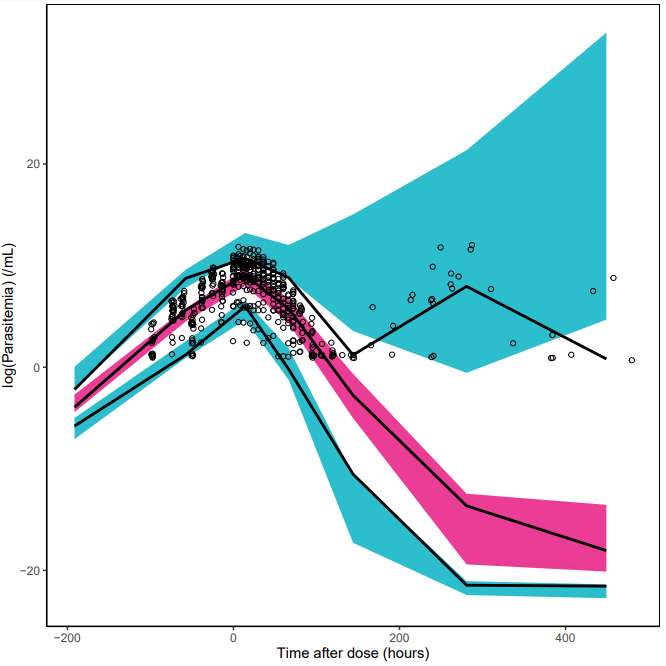

Supplement: Supplemental figures and tables — Supplemental material 1–4. [file aac.00891-23-s0001.docx]
